# Modern Electromagnetic-Radiation-Shielding Materials Made Using Different Knitting Techniques

**DOI:** 10.3390/ma17133052

**Published:** 2024-06-21

**Authors:** Zbigniew Mikołajczyk, Iwona Nowak, Łukasz Januszkiewicz, Monika Szewczyk, Joanna Junak

**Affiliations:** 1 Textile Institute, Faculty of Material Technologies and Textile Design, Lodz University of Technology, 90-924 Łódź, Poland; iwona.nowak@p.lodz.pl (I.N.); 224194@edu.p.lodz.pl (M.S.); joanna.junak@p.lodz.pl (J.J.); 2 Institute of Electronics, Faculty of Electrical, Electronic, Computer and Control Engineering, Lodz University of Technology, 90-924 Łódź, Poland; lukasz.januszkiewicz@p.lodz.pl

**Keywords:** knitted electromagnetic wave shields, shielding measurements, knitted fabric, shielding effectiveness

## Abstract

This paper summarizes the possibility of employing knitted textile barriers as a shield against electromagnetic fields to protect the human body from their negative impact. Ten variants of knitted fabrics made of electrically conductive yarns, steel, and copper wire that differed in stitch pattern, structural parameters, and raw material, were designed, manufactured, and tested. The knitted fabrics produced differed in structural parameters, including course and wale density, surface density, thickness, thread length in the loop, wale and course take-up, volume cover factor, and surface porosity. These parameters were examined in accordance with the research methodology used in knitting. Barrier measurements were taken in the direction of the wales and in the direction of the courses for two frequencies of electromagnetic fields: 2–4 GHz and 4–7 GHz. It was observed that the shielding effectiveness of the manufactured materials depends on the structural parameters of the fabric, the stiches applied, and the type of yarn.

## 1. Introduction

The development of modern civilization and rapid progress in electronics have made our everyday life much easier; however, this has resulted in new threats, such as exposure to electromagnetic fields. Protecting people against electromagnetic radiation has been the subject of scientific research for many years, and numerous materials are being created to serve as electromagnetic shields [1,2,3].

An electromagnetic field is a physical field in which forces of an electromagnetic nature act on a physical object that has an electric charge. An electromagnetic field is a system of two fields: an electric field and a magnetic field. Natural sources of radiation include the magnetic fields of the Earth, the Sun, visible and invisible infrared light, solar winds, atmospheric discharges, and the human body. With the development of technology, in addition to natural radiation, artificial man-made radiation sources have appeared, including X-ray generators, nuclear reactors, electrical devices, radio and telecommunications systems, and power installations [4,5,6]. Maxwell’s theory proves that electromagnetic fields move through space at the speed of light as electromagnetic waves, which are a disturbance of the electromagnetic field resulting from the coupling of electric and magnetic fields. Electromagnetic radiation can be divided into two ranges, the first of which is ionizing radiation (high-frequency radiation), and the second is non-ionizing radiation, which transports too little energy to lead to ionization. The boundary between these two ranges lies between the spectrum of visible and ultraviolet radiation. Depending on the frequency, wavelength, and nature, the following are distinguished:

- Radio waves—waves with the lowest frequencies, ranging from 3 kHz to 3 THz, and, at the same time, with the largest wavelengths, exceeding 1 m;
- Microwaves—waves with a frequency in the range of 1–300 GHz, used in radar, radio, and satellite communication, mobile telephony, and GPS navigation. They reach wavelengths of 1 mm to 1 m;
- Infrared—radiation with a frequency of 300 GHz–400 THz, emitted by objects with a temperature higher than absolute zero, e.g., by the human body or the Sun. The wavelength ranges from 700 nm to 1 m;
- Visible light—radiation with a frequency of 400 THz–790 THz and wavelengths from 380 nm to 700 nm.
- Ultraviolet (UV)—radiation with a frequency of 790 THz–30 PHz. Software can allow one to photograph UV radiation. The Sun is the source of this radiation, and extensive exposure of the skin to UV radiation is harmful to health.

Electromagnetic radiation covers a very wide frequency range, since an electromagnetic wave can refer to long waves used in telecommunication, with the lowest frequencies, and gamma radiation, with the highest frequencies and shortest wavelengths. Due to this broad spectrum, it is not possible to generally describe the effects that radiation produces. The first form is very high frequency radiation, with the ability to knock out electrons and break chemical bonds. It is the most dangerous for humans because it can cause damage to genetic material and cancer, even in small doses. On the other hand, taking a high dose of ionizing radiation leads to radiation sickness and death [7,8,9,10,11,12]. As a result, dose limits have been introduced, with an effective dose of 1 mSv set as the acceptable limit for the general population within a calendar year. In the case of apprentices and people in a profession with exposure to ionizing radiation, the annual dose limit is much higher, amounting to 50 mSv, but it may not exceed 100 mSv over a 5-year period. Pregnant women, on the other hand, should not be exposed to conditions in which the fetus could receive an effective dose that exceeds 1 mSv [13,14]. The second type of electromagnetic radiation is non-ionizing radiation, which has a vibration frequency below 300 GHz and carries too little energy to produce an ionizing effect on the body. The effects of low-frequency radiation are far less dangerous than those caused by ionizing radiation. Two types of effects of non-ionizing radiation can be distinguished: thermal and non-thermal. Thermal effects are associated with an increase in body temperature by 1 °C, while non-thermal effects occur when the temperature change does not exceed 1 °C; however, they are very difficult to recognize and describe due to the very small scale of these effects [15].

The negative impact of electromagnetic fields on the human body is well described in the literature [12,13,14,15]. The impact of radiation from mobile phones is of particular concern, as new technologies based on increasingly higher frequencies are constantly being introduced. From time to time, claims that telecommunication infrastructure and devices increase the risk of brain, head, and neck cancer tend to appear. Other disorders attributed to the impact of electromagnetic waves used in cellular technology are nervous system disorders, such as sleep disorders or insomnia, headaches and dizziness, and fatigue or impaired concentration [16,17,18].

The studies conducted to date have failed to confirm such a risk. However, it has been proven that radiation may disturb the speed of biochemical processes, in turn having a minimal impact on ion transport through cell membranes and potentially resulting in the production of small amounts of heat-shock proteins, affecting cell functioning. These changes are not, however, considered to be a direct threat to human health or life [10,11,12,13,14,15,16,17,18,19,20].

The International Agency for Research on Cancer, IARC, has developed a classification of carcinogenic substances and risk factors that indicates radiation emitted by mobile phones to be potentially dangerous [21,22,23,24,25,26,27,28]. Additionally, the classification states that radiation emitted by cellular networks belongs to a group of potentially cancer-causing agents [25,26,27,28,29]. Most indications of electromagnetic radiation having a negative impact derive from fertility research. Studies clearly indicate that sperm motility decreases as a result of radiation, but it was found that this is only one of many factors influencing infertility and not its direct cause [30]. Most studies on the effects of electromagnetic fields on fertility are conducted in men, but they also affect pregnant women. Studies show different effects caused by electromagnetic fields for both sexes. Studies of pregnant women indicate that the non-thermal effects of radiation may increase the incidence of spontaneous abortions [13,14]. Other studies have shown that a radiation dose of 4 Gy causes infertility in 30% of women. Due to the fact that pregnant women have a higher sensitivity to all kinds of dangerous factors, regulations have been introduced to control the employment of pregnant women who might be exposed to electromagnetic fields. Accordingly, pregnant women are not allowed to work in the range of electromagnetic fields with intensities exceeding the values for a safe zone [14,30].

In recent years, a lot of controversy has also been caused by the introduction of the 5G network, as the networks currently use frequencies in the range of 700 to 3600 MHz and can use frequencies up to 26 GHz. Again, specialists have assured us that the introduction of this network is harmless to the human body. There is also concern that the use of much higher frequencies in telecommunications will result in greater intensity of the above-mentioned symptoms. However, the relatively recent introduction of this network prevents the validation of exactly which symptoms occur as a result of the produced radiation.

Various methods have been adopted to suppress potentially harmful waves in order to protect human health and limit exposure to electromagnetic fields. Shielding is the most commonly used means of radiation protection. Electromagnetic shields are made of materials characterized by high conductivity, i.e., any raw material with electrically conductive ability can be used as an electromagnetic barrier, but with varying degrees of effectiveness. Electromagnetic shields can be made in two ways: first, by inserting a material that will scatter or absorb the electromagnetic field between the field source and the area where the field is to be lowered; second, by introducing additional field sources to lower the level of electromagnetic field in a specific area.

Electromagnetic field filtering is another important and frequently used means of radiation protection. The filters are usually used on power lines or the inputs and outputs of all kinds of signal lines and are designed to suppress electrical disturbances that flow to and from a device [31].

The appropriate type of electromagnetic field suppression depends on the situation and the place of use. Technological development means that there are increasingly more materials on the market for use as electromagnetic screens. The following are currently used in addition to the previously used sheets and meshes: metallic, metalized, and metal-containing materials; ferromagnetic, ferroelectric, ferrimagnetic, thin-film, and electrically conductive coatings; electrically conductive polymers; electrically conductive glass and transparent materials; electrically conductive paper, composites, nanomaterials, and metamaterials; and high-temperature superconductors [32]. As the power and frequency of devices that emit electromagnetic waves increase, so does the need to protect against their harmful radiation, for example, in the work environment. At this point, another problem arises: how to protect employees from harmful radiation so that they can freely complete their jobs. In such situations, textile products with properties that suppress electromagnetic radiation are the best solution. They are increasingly used as electromagnetic screens due to their low price, air and water permeability, and surface weight.

Electromagnetic barriers are made from textile materials by introducing electrically conductive elements into their construction. As a result, textile electroconductive barriers can be created using the following types of yarns: metalized; made of carbon fibers; containing metal fibers; possessing an electrically conductive core; or containing electrically conductive additives such as graphite, carbon black, metals, and semiconductors.

Shielding effectiveness (*SE)* is the measure of how effectively a material can attenuate electromagnetic fields. This can be expressed using the effective values of electric field intensity at the exposed side of the shield (*E*_1_) and the shielded side (*E*_2_), as in Equation (1) [33]:

(1) $$SE=20\cdot {log}_{10}\left(\frac{E_{1}}{E_{2}}\right)$$

The theoretical shielding effectiveness (*SE*) of a homogeneous material is typically described by three components: reflection losses (*R*), absorption losses (*A*), and secondary reflection losses (*B*), as in Equation (2):

(2) SE=R+A+B

The latter component (*B*) can often be neglected (*B* = 0) when the absorption losses exceed 8 dB, simplifying the *SE* to a two-component model. The literature explores this simplification further for both near-field and far-field scenarios. Since our study investigates high-frequency electromagnetic waves, far-field analysis is considered more appropriate. In this case, the reflection losses *R* are given in (3) and the absorption losses *A* in (4) [34]:

(3) $$R=168+10{log}_{10}\frac{\sigma }{\mu \cdot f}$$

(4) $$A=3.34\cdot 10^{-3}\cdot t\cdot \sqrt{f\sigma \mu }$$

where *σ* is electric conductivity, *μ* is magnetic permeability, and *f* is frequency.

Existing theoretical formulae for *SE* components are derived under the assumption of a solid conductor shield made of homogeneous material. In this paper, we investigate inhomogeneous material composed of non-magnetic fibers with different electric conductivity capabilities. While these formulae identify material conductivity and permeability as key factors that influence shielding properties, we focus on electrical conductivity as the primary parameter impacting the performance of the presented materials due to their specific characteristics.

The effectiveness of a shielding material depends on its conductivity: the higher the conductivity, the more effectively the material will reflect electromagnetic waves. The conductivity of selected materials varies; for example, silver has a conductivity of 6.3 × 10^7^ S/m, copper has 5.9 × 10^7^ S/m, gold has 4.5 × 10^7^ S/m, and steel has 5.0 × 10 S/m.

Work on textile shielding materials based on knitted fabrics, fabrics, and nonwoven materials exists in the literature [35,36,37,38,39]. The most commonly used raw materials with electromagnetic action are silver, nickel, copper, stainless steel, carbon, or materials that are a combination of these elements. Shielding materials can be produced by soaking fabric in a solution, producing materials with an EMI shielding of 340–80 dB (with four surfacing and drying cycles). Shielding materials can also be created by coating technology (nickel, Ag nanoparticles, carbon nanotubes, FE304 nanoparticles), delivering an EMI shielding of 30–68 dB in a single layer of fabric [35]. A column-knitted fabric was produced from silver-plated polyamide threads, with a shielding efficiency of *SE* 19 dB obtained at the test frequency of 2.0 GHz; however, it was shown that the shielding efficiency decreased after washing tests [36]. Most of the shielding textiles found around the world are materials in the form of woven or nonwoven fabrics. Knitted shielding fabrics also exist, but they are scarce. Fabrics have completely different structures and a high surface density, so it is difficult to compare them with knitted fabrics in terms of shielding properties. Shieldex, among others, sells fabric screens such as Bremen PW fabric, which has a shielding efficiency of 67 dB when measured at the frequency of 0.2–14 GHz; Berlin RS fabric, with a shielding efficiency of 65 dB; Nora Dell fabric, with a shielding efficiency of 84 dB; Bremen RS fabric, with a shielding efficiency of 62 dB; Pisa BW fabric, with a shielding efficiency of 82 dB; and Zell PW CR fabric, with a shielding efficiency of 80 dB at a frequency of 0.2–10 GHz. The company also offers heat-welded nonwovens, e.g., Bonn (shielding efficiency of 63–78 dB at 0.2–14 GHz) [40]. The company Marktek Inc. offers shielding fabrics such as EaonTex, with a surface resistivity of 8–102 ohm/m^2^ [41]; no data on the shielding efficiency of this fabric were found.

When choosing a material for an electromagnetic screen, it is important to consider that metal materials degrade during use due to factors such as temperature and humidity [42].

The tentative facts posited on the negative impact of electromagnetic waves on the human body inspired the authors of this paper to undertake research on the technology of knitted elastic electromagnetic screens that are used in clothing for people requiring special care, including newborns and children, pregnant women, and seniors. The overarching scientific objective of this research was to analyze the influence of the structural and electrical parameters of shielding knitted fabrics on the effects of electromagnetic wave attenuation. The knitted materials produced in this work can be used in everyday clothing or protective clothing, as well as in home accessories such as blankets, rugs, and curtains.

The cost of producing a shielding knitted fabric from electrically conductive yarn is slightly higher than the cost of producing a traditional knitted fabric made of cotton or polyester yarn; however, the high stakes of human health protection designate this cost as less important.

## 2. Materials and Methods

### 2.1. Characteristics of the Produced Knitted Fabrics

For research purposes, nine variants of weft-knitted fabrics with plain stitches and one warp-knitted sample were constructed from electroconductive yarn. The first four variants were manufactured using multi-filament silver-plated thread from Shieldex, with a linear density of 150 dtex. Variant 5 was a plain-weave fabric made of silver-plated Shieldex yarn in addition to synthetic fibers with a linear density of 340 dtex. Variant 6 was a two-layer knitted fabric with a face made of silver-plated Shieldex yarn in addition to synthetic fibers, 340 dtex, and a back made of cotton yarn, 180 dtex (plated knitting fabric, polyamide). Variant 7 was made of silver-plated yarn from Amann in addition to synthetic fibers, and it had a linear density of 120 dtex (polyester and polyamide fibers). A plain-stitch knitted fabric made of silver-plated Shieldex yarn with a linear density of 600 dtex was used to make variant 8.

**Figure 1:**
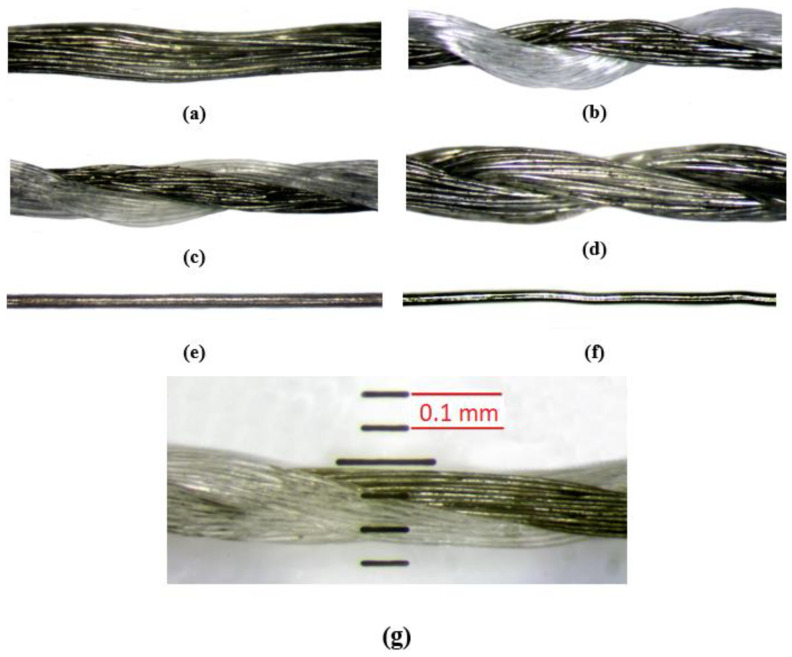
Microscope thread images: (**a**) Shieldex, 150 dtex; (**b**) Shieldex, 340 dtex; (**c**) Amman, 120 dtex; (**d**) Shieldex, 600 dtex; (**e**) Cu wire; (**f**) steel wire, (**g**) scale for sample yarn.

Variant 9 was made of steel wire with a diameter of 0.063 mm. A double-needle comb warp-knitted fabric composed of tricot and velvet stitches made of copper wire with a diameter of 0.073 mm was used for variant 10. The yarns are shown in Figure 1.

The first nine variants of the knitted fabrics were made on circular weft-knitting ma-chines with needle gauges E14 and E20, while variant 10 was produced on a double-needle comb warp-knitting machine, gauge E28.

**Figure 2:**
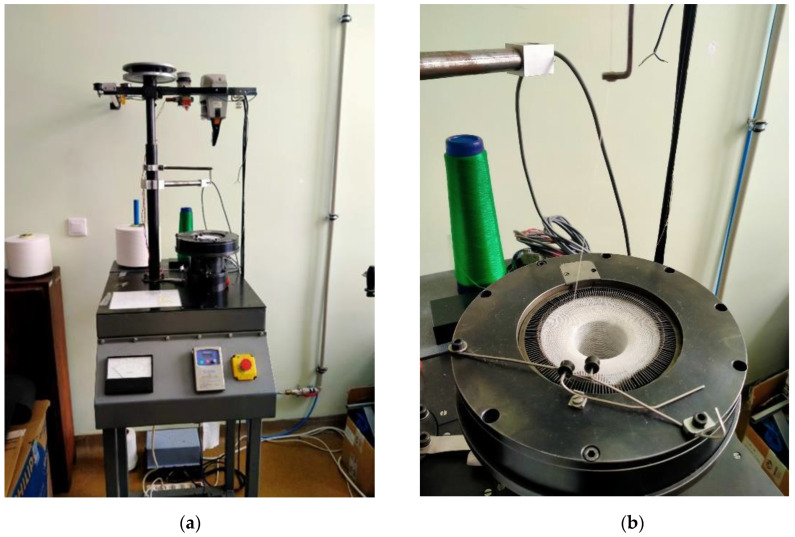
Single-bearing cylindrical crochet machine (cylinder diameter = 4″; needle number *NE* = 14; number of needles, *L* = 169; number of revolutions, n = 50–200/min). (**a**) Full view of the machine; (**b**) view of the cylinder.

**Figure 3:**
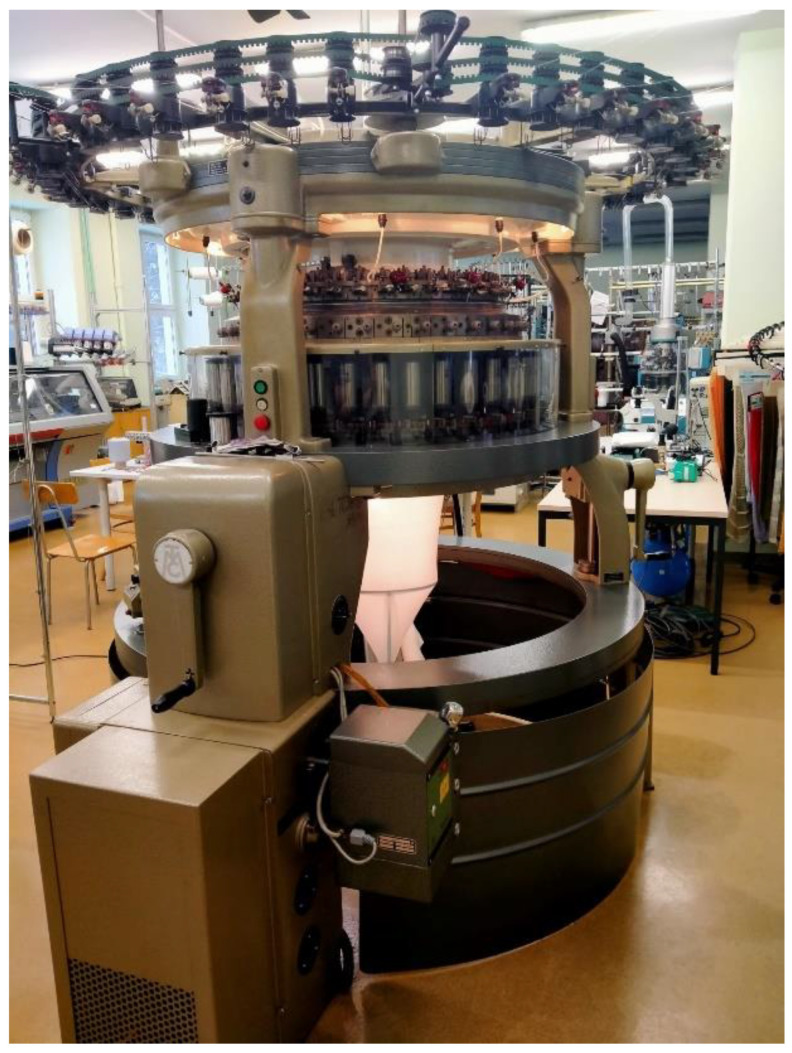
Cylindrical double-bearing crochet machine from Mayer & Cie (cylinder diameter = 30″; gauge pitch = 1.27 mm; needle number, *NE* = 20; number of needles, *L* = 2 × 1872).

**Figure 4:**
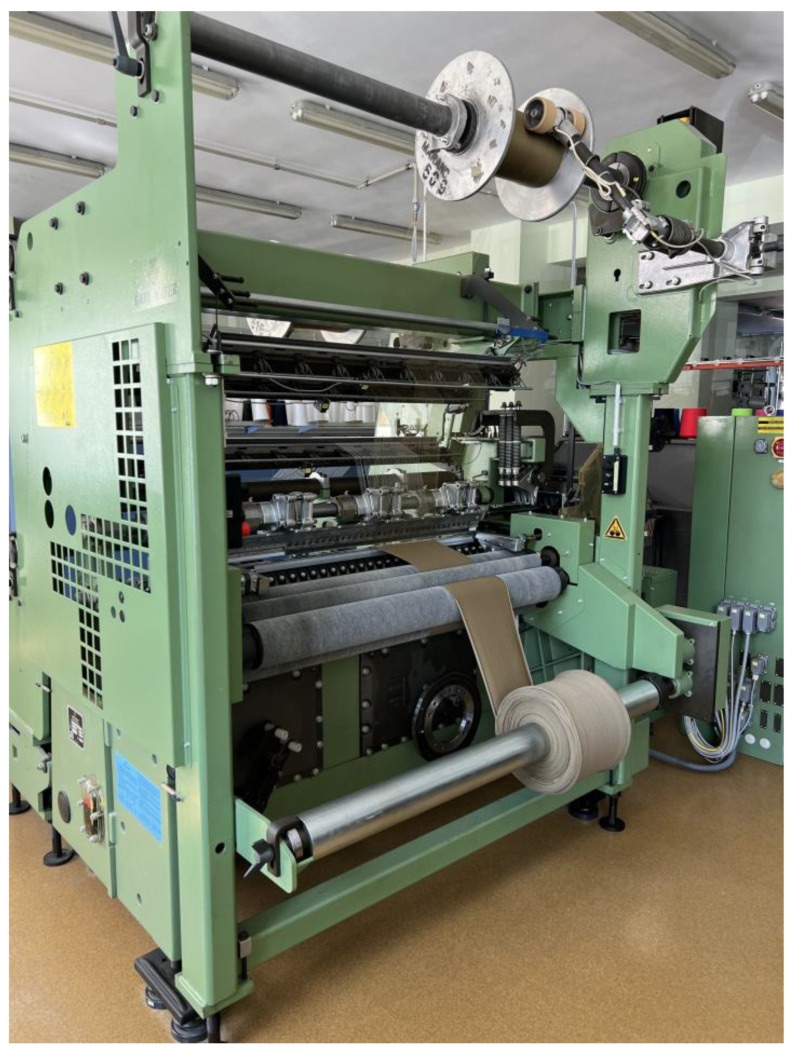
The HKS 3 warp-knitting machine from Karl Mayer, with needle number *NE* = 28.

The knitting machines used in this work are presented in Figure 2, Figure 3 and Figure 4.

The parameters of the knitted fabrics produced are given in the next section.

### 2.2. Characteristics of Methods for Measuring Knitted Fabric Parameters

**Figure 5:**
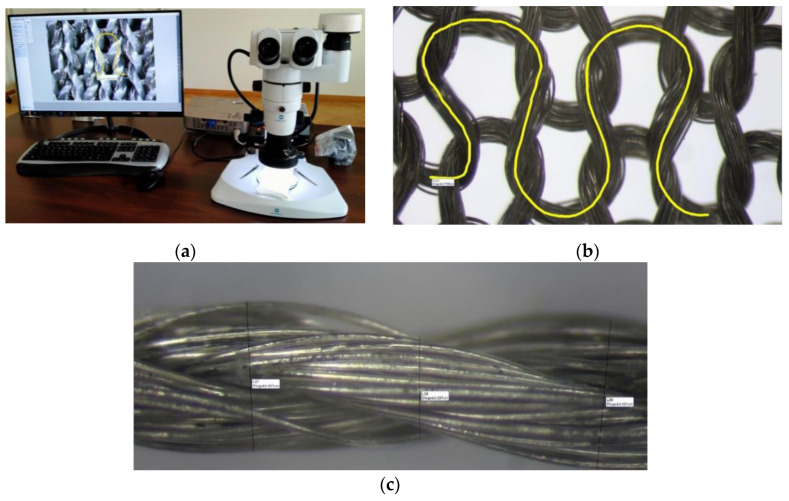
(**a**) Measurement station equipped with a microscope and view system; (**b**,**c**) examples of determining the length of yarn in a stitch. (A dozen or so points lying on the yarn axis in the loop were manually marked. The program then automatically connected the dots and approximated them in the form of a curve. After determining the yarn axis, the result of the thread length in the stitch was shown).

Thread thickness was determined by analyzing images of the knitted fabrics using the Opta View program Version 4.3.0.6001. This software works with the OPTA-TECH SN series stereoscopic microscope (Figure 5a), which is equipped with a digital camera. The program was used to digitally photograph the structure of a knitted fabric and measure its structural parameters—in this case, the length and thickness of the thread in the mesh. The software allowed us to take photos and videos which could then be subjected to graphic processing such as brightness, sharpness, contrast, and color correction, as well as resizing and rotating photos. An additional advantage of the software is its ability to perform measurements on a captured image, allowing for measurement of the distances between points, the angle created by two intersecting lines, and the area of a selected shape. This option was used to measure the yarn diameter and the length of the yarn in the mesh. To determine the length of the yarn in the eye, it was necessary to calibrate the device, select the measurement option, and then, draw a line along the axis of the yarn in the eye. In this way, the length of several stitches of the knitted fabric was measured and an average was taken from the obtained value. The thread thickness was measured in a similar way. Measurements were taken in several of the widest and narrowest parts of the thread, and then, averaged, denoting the thread thickness. Due to the experimental method of measurement, the obtained values may differ slightly from the actual values (Figure 5b,c).

**Figure 6:**
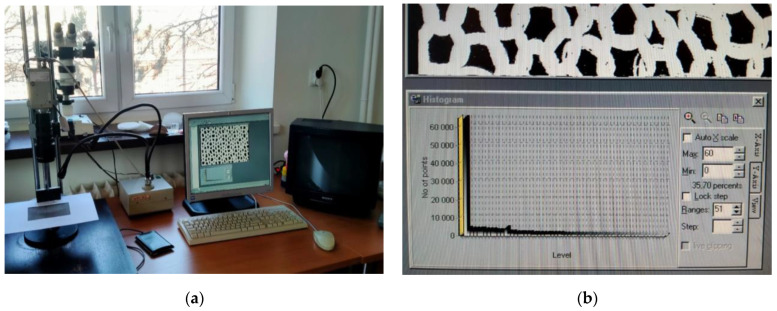
(**a**) Measuring station for determining experimental porosity; (**b**) histogram made by the TEXTIL-STUDIO program.

The surface porosity coefficient was determined using an optoelectronic method that included a computer network, with the TEXTIL-STUDIO computer program being utilized for this purpose. The measurement station (Figure 6a) was equipped with a WV-CP Panasonic digital CCTV camera with an image converter, lighting, a computer equipped with the TEXTIL-STUDIO program, and a TV screen. We determined the surface porosity coefficient using a measurement station made by taking photos of the knitted fabric.

**Figure 7:**
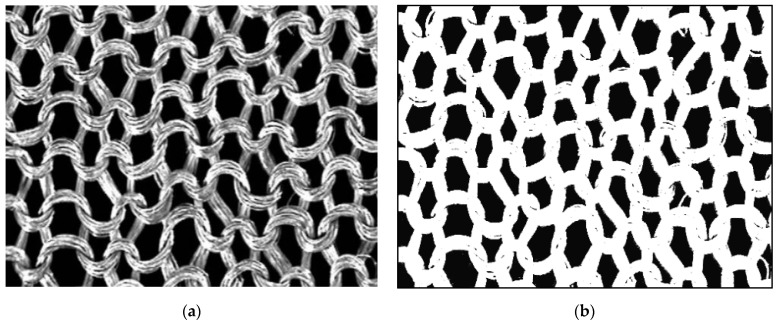
(**a**) Actual view of the knitted fabric; (**b**) binary image of knitted fabric.

The image recorded by the camera was visible on the TV screen and in the TEXTIL-STUDIO program window. The program allowed us to set the desired image acquisition characteristics, i.e., contrast and luminance. After selecting the parameters, a photograph was taken, and then, transformed into a binary image of the knitted surface, using the histogram function. This function recorded the number of black and white pixels. The black pixels correspond to the background of the binary image and the white ones correspond to the yarn. The number of pixels could be manipulated to obtain the best possible reflection of the real image in the binary image. For this purpose, we used the functions “Max” and “Min” shown in the image below (Figure 6b). When a clear image of the knitted fabric and its background was obtained, the fill factor value was read. In this case, background filling was determined, so the obtained value was subtracted from 100 and expressed as a percentage to denote the surface porosity of the knitted fabric. Figure 7 shows an example photo of a knitted fabric and its binary image (white color—knitted fabric; black color—porosity).

### 2.3. Shielding Effectiveness Testing Methodology

Defining reliable test procedures to measure the shielding effectiveness (SE) over a wide frequency range, namely, between a few kilohertz and a few tens of gigahertz, has arisen as a crucial challenge due to research on new advanced shielding materials, thin conducting films, and nanomaterials, as well as the increasing operating frequencies of devices and systems. Various methods of measuring the *SE* of flat material samples against far-field or near-field sources have been proposed throughout the years, including waveguide test fixtures, anechoic chambers, or, more recently, nested reverberating chambers [43,44,45,46,47,48,49,50,51,52]. The usage of single-coaxial transverse electromagnetic (TEM) cells in accordance [50] or [51] are well-known approaches for measuring the *SE* of isotropic planar materials against plane waves with normal incidence. In the first approach, a test sample is placed in a coaxial waveguide with a continuous inner conductor. In the second test fixture, a flanged coaxial transmission line holder with an interrupted inner conductor is used.

**Figure 8:**
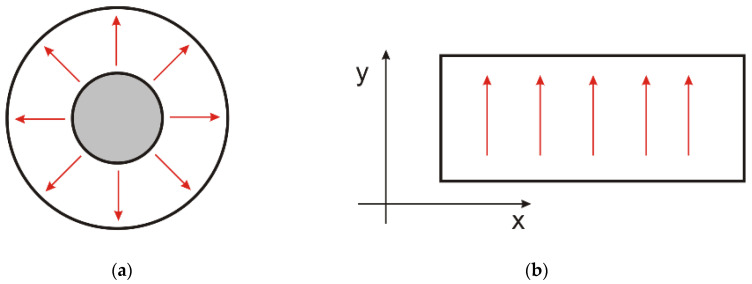
Electric field orientation in waveguides: (**a**) coaxial; (**b**) rectangular in fundamental mode.

Coaxial fixtures operate in TEM mode, in which the electric field intensity has a radial distribution and is perpendicular to the magnetic field of azimuthal orientation (see Figure 8a). For this reason, the TEM line method is suitable for characterizing homogeneous materials, as their shielding properties do not depend on the direction of the electric field. The knitted fabrics made of the conductive yarns used in this study are not homogeneous materials. Due to the regular arrangement of the yarns (the wales and courses), this type of material has different shielding properties depending on the orientation of the electric field in relation to the conductive fibers. In order to identify how the orientation of the electric field relative to the rows and columns influences the shielding effectiveness, the method presented below was applied. We employed a rectangular waveguide in the fundamental mode (TE 10). In this mode, the electric field was parallel to the shorter side of the waveguide cross-section (y-direction in Figure 8b) and perpendicular to the direction of the wave propagation. A sample material was placed inside the two sections of the rectangular waveguide. The shielding effectiveness was obtained from the wave attenuation measured between the input and output of the waveguide. By changing the orientation of the test sample in relation to the direction of the electric field, it was possible to determine the dependence of the shielding effectiveness on the direction of field.

**Figure 9:**
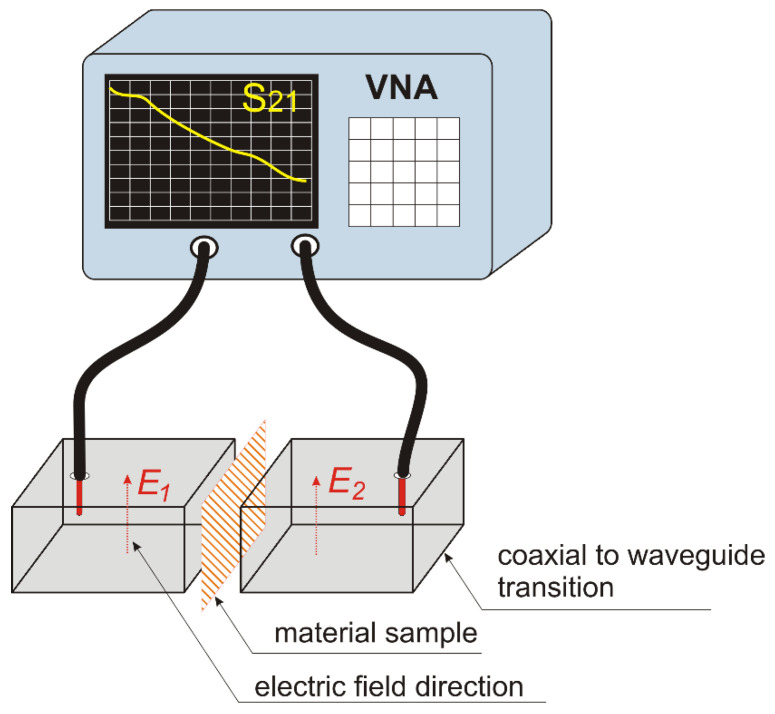
Measurement setup.

The measurement setup presented in Figure 9 was used. The shielding effectiveness (*SE*), in decibels, was defined as the ratio of the magnitude of the incident electric field, *E*_1_, to the magnitude of the electric field, *E_2_*, transmitted through the material (Equation (1)) [52].

During the measurements, attenuation of the electric field caused by the tested material increased the attenuation of the signal between the two rectangular waveguides. In order to measure the attenuation with a vector network analyzer (VNA), we used coaxial-to-waveguide transitions. A vector circuit analyzer was connected to coaxial transitions using measuring cables, as shown in Figure 9. The system attenuation (S21 parameter) was measured in the range of the frequencies transmitted by the waveguides. The shielding efficiency was calculated as the difference between the system attenuation without the sample compared to the value with the material sample.

**Figure 10:**
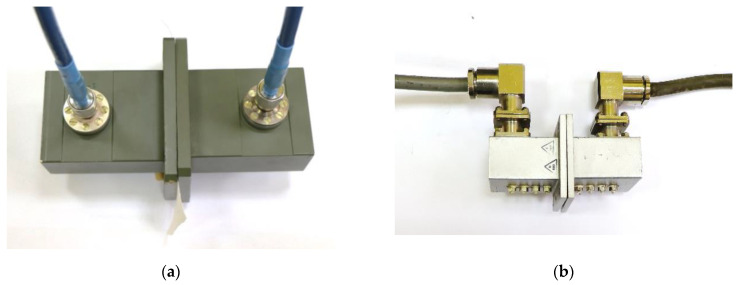
(**a**) The WR284 waveguide; (**b**) the WR159 waveguide.

In our study, we used electromagnetic waves with a wide frequency range of 2 to 7.4 GHz to investigate shielding effectiveness. This range was chosen because it covers the frequencies used in popular wireless systems, including 4th-generation mobile radio communication systems such as the LTE system, which uses the 2.1 GHz band. Additionally, popular 5th-generation (5G) systems use the 3.3–3.8 GHz and 3.7–4.98 GHz bands (outside Europe). The considered frequency range also includes the unlicensed ISM bands (2.4, 5.8 GHz), where the radiation density is high due to the popularity of wireless devices. The 6 GHz band is used by the latest Wi-Fi networks and is expected to become more popular in the near future. In order to measure the properties of the materials over a wide frequency range, two sets of waveguide transitions were used. The first waveguide used was WR284 as shown in Figure 10a. It had a lowest-order-mode cutoff frequency equal to 2.078 GHz, and its upper-mode cutoff frequency was 4.156 GHz. Its dimensions were 72.136 mm × 34.036 mm. The second waveguide used was WR159 as presented in Figure 10b. It had a lowest-order-mode cutoff frequency equal to 3.712 GHz, and its upper-mode cutoff frequency was 7.423 GHz. Its dimensions were 40.386 mm × 20.193 mm. Using these elements, it was possible to characterize the materials in a frequency range from 2.08 GHz to 7.4 GHz.

For each fabric variant, measurements were taken in both frequency bands in the direction along the wales and courses. While measuring the electromagnetic wave along wales, the fabric was arranged between the waveguides so that the wales were parallel to the shorter side of the waveguide, i.e., in the direction of wave propagation. While taking measurements along the courses, the fabric was arranged in the opposite way. The basic parameter defining the shielding properties of a given material is the shielding effectiveness (*SE*). It is an indicator of the electromagnetic field weakening at a specific point in space due to the introduction of a shielding material between this point and the field. In the case of the tested knitted fabrics, shielding effectiveness was expressed in decibels, a unit commonly used in radio engineering and telecommunications.

## 3. Results

**Figure 11:**
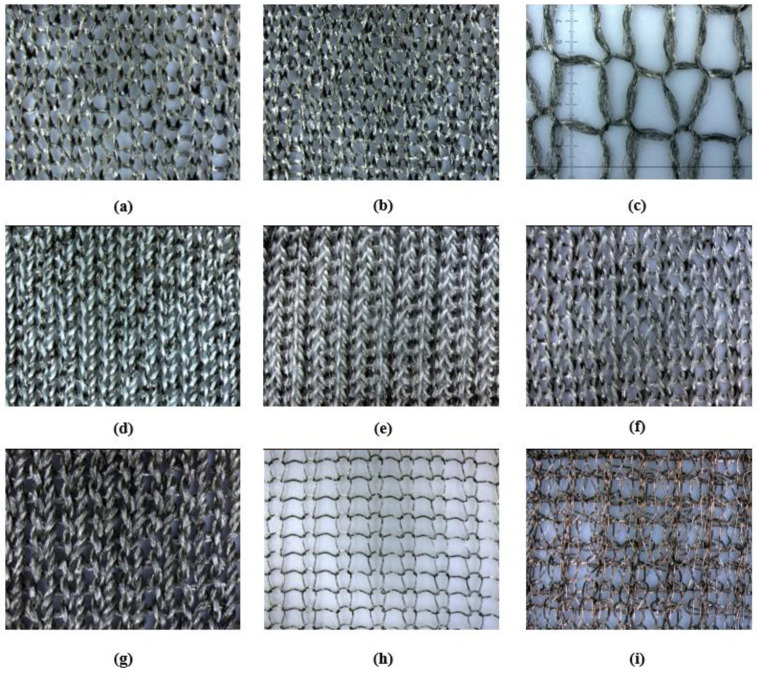
Microscope images of knitted fabrics: (**a**) knitted fabric 1 (variant 1); (**b**) knitted fabrics 2 and 3; (**c**) knitted fabric 4; (**d**) knitted fabric 5; (**e**) knitted fabric 6; (**f**) knitted fabric 7; (**g**) knitted fabric 8; (**h**) kitted fabric 9; (**i**) knitted fabric 10 (magnification 4.4×).

The produced knitted fabrics differed in structural parameters, including course and wale densities; surface density; fabric thickness; thread length in the loop; take-up coefficients; and linear, surface, and volume cover factors. The measured parameters of the produced knitted fabrics are summarized in Table 1, while Figure 11 presents photographs of the fabrics with different loop densities (fabric face).

No difficulties were encountered in processing the metalized thread during the production process.

As seen in the microscopic photos (Figure 11), the produced knitted fabrics differ in mesh size, porosity, fill factor, and the type of weave used, among others. Detailed data on the knitted fabrics can be found in Table 1.

**Figure 12:**
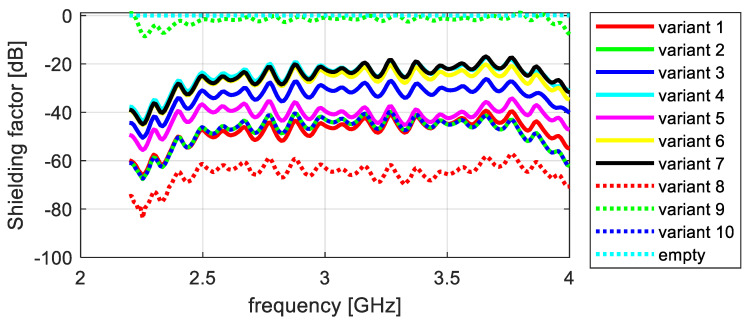
Attenuation along courses, frequency range 2–4 GHz.

**Figure 13:**
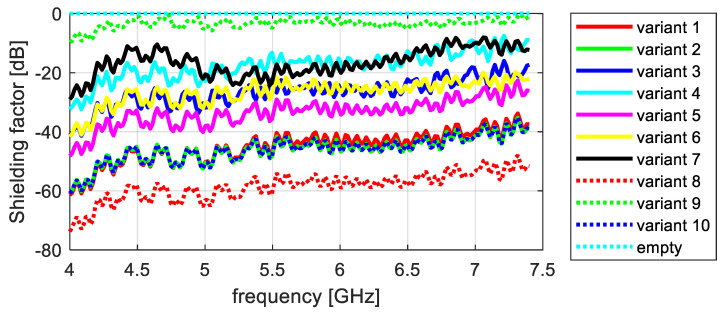
Attenuation along courses, frequency range 4–7 GHz.

**Figure 14:**
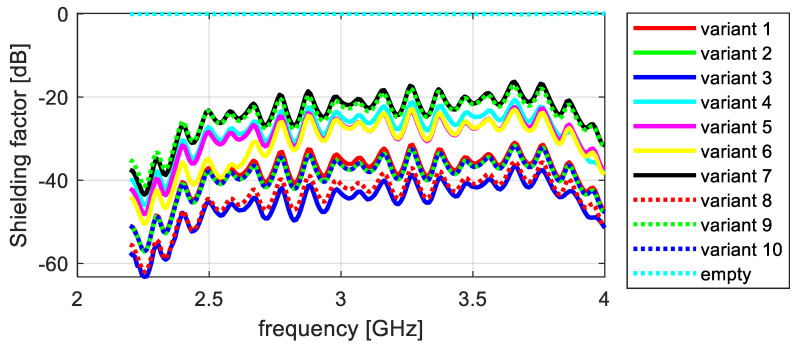
Attenuation along wales, frequency range 2–4 GHz.

**Figure 15:**
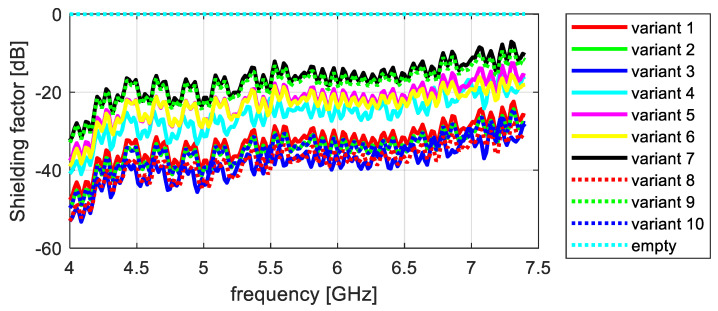
Attenuation along wales, frequency range 4–7 GHz.

The shielding effectiveness measurements of the tested knitted fabrics differ significantly, depending on the sample’s structural parameters. To illustrate the differences observed in the attenuation properties between the tested variants, they were summarized in a graph. In this way, four partial graphs were created, showing successive measurements of electromagnetic field attenuation in the frequency ranges of 2–4 GHz and 4–7 GHz, taken along the wales and courses (Figure 12, Figure 13, Figure 14 and Figure 15).

## 4. Discussion

The manufactured knitted fabric variants are characterized by loop densities ranging from 928 to 15,553 loops/dm^2^. The thickness of each sample lies between 0.28 and 1.38 mm. The yarn length in the loop also varies, from 3.08 to 11.14 mm. The surface porosity of the tested variants ranges from 10 to 824.6%, surface mass from 15.5 to 369.2 g/m^2^, and surface resistance from 0.3 Ω to 2.4 kΩ.

The shielding effectiveness depends on the material variant and the material’s orientation towards the electric field vector. Based on an analysis of the data presented in Figure 9, which show attenuation along courses in a frequency range of 2–4 GHz, it can be concluded that variant 9 had the lowest shielding effectiveness, with an average shielding factor value of −1.8 dB. The highest shielding effectiveness was exhibited by variant 8, with an average shielding factor value of −65 dB. Variants 1, 2, and 10 had very similar average values of around −47 dB. Variant 3 showed slightly worse performance, with an average shielding factor of −33 dB. Variants 4, 6, and 7 attenuated EM waves to a lesser extent, with average values of around −25 dB.

The results obtained for the higher frequency range (4–7.5 GHz) and the same electric field orientation are similar to those obtained for the 2–4 GHz range. Based on an analysis of the data presented in Figure 10, it can be concluded that variant 9 had the lowest shielding effectiveness, with an average shielding factor value of −3.4 dB in this range. Variant 8 had the highest shielding effectiveness in this range, with an average shielding factor value of −58 dB; however, this was lower than the average value for the 2–4 GHz range. Variants 1, 2, and 10 had very similar average values of around −46 dB. Variant 5 was slightly worse, with an average shielding factor of −33.4 dB. Variants 3 and 6 had an average shielding factor value of −26.5 dB. Variants 4 and 7 attenuate EM waves to a lesser extent, with average values of around −17 dB.

The shielding factors of the different material variants measured with the electric field oriented along the wales (Figure 11 and Figure 12) are more similar to each other than the measurements along the courses. Based on an analysis of the data presented in Figure 11, which were obtained in the frequency range of 2–4 GHz, it can be observed that variants 7 and 9 had the lowest shielding effectiveness, with average shielding factor values of −24 dB in this range. In this range, variant 3 had the highest shielding effectiveness, with an average shielding factor value of −45 dB. Variant 8 showed an average value of −44 dB. Variants 1, 2, and 10 had very similar average values of around −39 dB. Variant 6 was slightly worse, with an average shielding factor of −31.3 dB. Variants 4 and 5 had an average shielding factor value of −29 dB.

Similarly, the material variants can be ranked for the higher frequency range and the same electric field orientation. Based on an analysis of the data presented in Figure 12, which were obtained in the frequency range of 4–7.5 GHz, it can be observed that variants 7 and 9 had the lowest shielding effectiveness, with an average shielding factor value of −17 dB. Variant 3 had the highest shielding effectiveness in this frequency range, with an average shielding factor value of −38 dB. Variant 8 also showed an average value of −37 dB. Variants 1, 2, and 10 had very similar average values of around −34 dB. Variant 4 was slightly worse, with an average shielding factor of −25 dB. Variants 6 and 5 had an average shielding factor value of −23 dB.

The obtained results prove that all of the manufactured knitted fabric variants attenuate electromagnetic waves over the entire range of tested frequencies. As wave frequency increased, a slight decrease in shielding effectiveness could be observed, which was especially visible for the high frequencies. Attenuation capacity also varied for different fabric orientations in relation to the waveguides (direction along the wales and along the courses).

For the measurements carried out along the wales in the full frequency range from 2 to 7 GHz, the highest shielding effectiveness was observed for variants 3 and 8. For 2–4 GHz, their shielding efficiency was 63 dB, and, for 4–7 GHz, it was 54 dB. Variant 3 was made from a single synthetic Shieldex yarn with a surface density of 150 dtex, and variant 8 was from a combined Shieldex thread of 600 dtex.

For the shielding effectiveness measurements taken along the courses in the frequency range of 2–4 GHz, the best result of 84 dB was obtained for variant 10, which was made of copper wire with a diameter of 0.0073 mm; in the frequency of 4–7 GHz, the maximum value of 73 dB was achieved by variant 8. The obtained results are very good and comparable to, or even better than, the textile shielding materials commercially sold by Shieldex.

The deterioration in shielding properties with increasing frequency is an expected phenomenon for the type of shielding material considered. In the case of conductive electromagnetic shields, efficiency decreases with the introduction of holes in the shielding material [53]. Each hole can act as a secondary source of radiation in the form of a parasitic slot antenna, through which electromagnetic wave energy passes to the other side of the shield. However, this phenomenon is very complex and depends on many factors such as the geometry of the holes and the material parameters of the shield. In the case of knitted fabrics, the material is not solid and consists of many irregular holes. Thus, the phenomenon also occurs in this material. Thorough investigation requires computer simulations of the knitted fabric structures and will be the next stage of our work.

Analysis of the interaction between the materials described in this article and electromagnetic waves is far more complex than for the solid homogeneous materials described in the literature [35]. In our case, answering the question of the relationship between the knit’s structure and its shielding properties requires the development of a numerical model and computer analysis of this complex system [54]. Considering the conductive fibers that we used, the dominant mechanism will be the induction of electric current in the knit, the distribution of which results from both the properties of the fibers and the parameters of the weave as well as the frequency of the EM wave. A different degree of field shielding is observed depending on the conductivity of the fibers, their spatial arrangement, and the quality of the electrical contact between them. Since our measurements showed material anisotropy (shielding level dependent on the orientation of the electric field), it is probable that the dominant mechanism responsible for shielding was the induction of currents along courses. We hope that our further research will allow us to answer this question.

The obtained research results are quite promising in view of further design and modeling projects aimed at optimizing the barrier properties of knitted fabrics intended for protective clothing for people with specific needs.

## 5. Conclusions

The research findings support multiple conclusions about the electromagnetic wave attenuation properties of the manufactured knitted material variants:

1. Effectiveness across frequency ranges: This research demonstrated that all of the knitted fabric variations efficiently attenuated electromagnetic waves over the entirety of the utilized frequency ranges. However, a modest decrease in shielding effectiveness was noted with the increase in frequency; this was especially apparent at the highest band (7 GHz).
2. Importance of fabric features: Each fabric’s properties, including surface porosity, loop density, and yarn length in the loop, affected the shielding capacity. The shielding performance was further affected by variations in fabric orientation with respect to the waveguides (along the wales and along the courses).
3. Cloth composition: The kind of yarn or thread used in the cloth, along with its overall composition, affected how successful the shielding was. Variants using integrated Shieldex thread or yarn demonstrated encouraging outcomes, particularly at lower frequencies.
4. Fabric orientation: Shielding performance was impacted by each fabric’s orientation with respect to the waveguides. When assessed along the courses or along the wales, certain versions demonstrated the best shielding performance.
5. Prospective uses: The findings of this study have implications for the creation of knitted materials that are better suited for electromagnetic shielding, especially for protective gear meant for people with special requirements. These results can be expanded upon in future design and modeling studies to improve the barrier qualities of knitted materials for a range of uses.

Knitted materials have enormous potential for use in shielding applications in a variety of fields, such as electronics, healthcare, and aerospace. These adaptable textiles can be used to create protective apparel that will shield both workers and users from electromagnetic radiation. Their versatility can also be seen in the automotive and architecture industries, where they are used to reduce electromagnetic interference and improve comfort and safety. The materials presented in this article have many potential applications, and their development, as well as improvement in methods for measuring their properties, is an important research direction.

## Figures and Tables

**Table 1:** (a,b) Performance parameters of the shielding knitted fabrics.

(**a**)							
**Knitted** **Fabric Variant**	**Course** **Density, *Pr* (course/10 cm)**	**Wale Density, *Pk* (wales/10 cm)**	**Loop Density, *Prk*** **(loops/1 dm^2^)**	**Loop Shape Factor, *C***	**Fabric** **Thickness (mm)**	**Loop Length, *lr* (mm)**	**Wale Take-Up,** ***Wk***
1	151	103	15,553	0.72	0.45	3.08	3.18
2	129	91	11,739	0.73	0.40	3.78	3.44
3	74	68	5032	0.92	0.38	5.03	3.42
4	32	29	928	0.91	0.28	11.14	3.23
5	138	106	14,628	0.78	0.64	3.18	4.54
6	142	99	14,058	0.69	1.38	3.22	4.60
7	111	94	10,434	0.85	0.48	3.91	4.34
8	91	74	6734	0.69	0.86	5.40	4.90
9	133	66	8778	0.50	0.60	3.74	4.99
10	98	120	11,760	1.23	0.67	Velvet 6.50 Tricot 4.30	Velvet 6.31 Tricot 4.17
(**b**)							
**Knitted** **Fabric Variant**	**Course Take-Up, *Wr***	**Surface** **Mass, *Mp* (g/m^2^)**	**Linear Cover Factor, *Zl*** **(loops/1 dm^2^)**	**Surface Cover Factor, *Zp***	**Volume Cover Factor, *Zo***	**Surface** **Porosity** **(%)**	**Surface** **Resistance** **(Ω/10 cm)**
1	4.40	84.33	13.39	0.96	2.22	64.3	1.3
2	4.73	67.25	16.43	1.01	2.36	59.6	1.8
3	3.73	47.50	21.87	1.72	3.58	41.4	1.8
4	3.56	15.50	48.44	4.21	6.51	29.9	3.4
5	3.53	201.15	12.72	0.79	2.58	82.0	3.2
6	3.19	324.09	12.88	0.88	6.18	84.6	5.2
7	3.69	127.70	19.55	1.22	3.73	65.5	2.9
8	3.38	239.85	14.59	0.88	2.61	81.3	0.3
9	2.49	96.03	63.33	5.01	63.86	10.0	2000.0
10	Velvet 7.83 Tricot 5.18	369.02	Velvet 89.04 Tricot 58.90	1.08	64.91	35.1	2.4

## Data Availability

The raw data supporting the conclusions of this article will be made available by the authors on request.

## References

[1] Ongel K., Gumra N., Ozguner F. (2009). The Potential Effects of Electromagnetic Field: A Review. Cell Membr. Free Radic. Res..

[2] World Health Organization (2002). Establishing a Dialogue on Risks from Electromagnetic Fields.

[3] Chen H., Lee K.C., Lin J.H., Koch M. (2007). Fabrication of conductive woven fabric and analysis of electromagnetic shielding via measurement and empirical equation. J. Mater. Process. Technol..

[4] Lamza Ł. (2019). Electromagnetic Field and Man.

[5] Białoszewski P. (2007). Electromagnetic Field in the Environment—Description of Sources and Research Results.

[6] Olsen N., Hulot G., Lesur V., Finlay C., Beggan C., Chulliat A., Sabaka T.J., Floberghagen R., Friis-Christensen E., Haagmans R. (2015). The Swarm Initial Field Model for the 2014 geomagnetic field. Geophys. Res. Lett..

[7] Rydzyński K. (2018). Electromagnetic Impact of Millimeter Waves on the Health of 5G Network Project Workers and the General Population.

[8] Knittel D., Schollmeyer E. (2009). Electrically high-conductive textiles. Synth. Met..

[9] Ching I.S., Jin T.C. (2004). Effect of stainless steel-containing fabrics on electromagnetic shielding effectiveness. Text. Res. J..

[10] Zhang Y., Li B., Liu S., Hao W. (2012). Electromagnetic wave absorption properties and mechanical properties of aramid fiber reinforced cement. Adv. Mater. Res..

[11] Wieckowski T., Jankukiewicz M. (2006). Methods for evaluating the shielding effectiveness of textiles. Fibers Text. East. Eur..

[12] White D. (1971). A Handbook Series on Electromagnetic Interference and Compatibility.

[13] Wdowiak A., Mazurek P. (2016). The Influence of Electromagnetic Fields on Human Reproduction. Electrotech. Insp..

[14] Serdyńska M., Pawelczyk L., Jędrzejczak P. (2008). Epidemiology of Infertility.

[15] Lerchl A., Klose M., Grote K., Wilhelm A.F., Spathmann O., Fiedler T., Streckert J., Hansen V., Clemens M. (2015). Tumor promotion by exposure to radiofrequency electromagnetic fields below exposure limits for humans. Biochem. Biophys. Res. Commun..

[16] Magiera A., Olecka I. (2019). Mobile Telephony and Its Impact on Human Health.

[17] Shyr T.W., Shie J.W. (2012). Electromagnetic shielding mechanisms using soft magnetic stainless steel fiber enabled polyester textiles. J. Magn. Magn. Mater..

[18] Singh K., Nagaraj A., Yousuf A., Ganta S., Pareek S., Vishnani P. (2016). Effect of electromagnetic radiations from mobile phone base stations on general health and salivary function. J. Int. Soc. Prev. Community Dent..

[19] Agarwal A., Desai N., Makker K., Varghese A., Mouradi R., Sabanegh E., Sharma R. (2009). Effects of radiofrequency electromagnetic waves (RF-EMW) from cellular phones on human ejaculated semen: An in vitro pilot study. Fertil. Steril..

[20] Albertson IV R.T., Arthur J., Rashid M.H. Overview of electromagnetic interference. Proceedings of the 2006, 38th Annual North American Power Symposium.

[21] Ting-Ting L., Rui W., Ching-Wen L., Mei-Chen L., Jia-Horng L. (2013). Manufacture and Effectiveness Evaluations of High-Modulus Electromagnetic Interference Shielding/Puncture Resisting Composites. Text. Res. J..

[22] Yakymenko I., Mor O., Tsybulin O., Ya K., Kyrylenko S., Sidorik E. (2015). Subjective symptoms in young cell phone users in Ukraine. Environ. Health.

[23] Tsybulin O., Sidorik E., Brieieva O., Buchynska L., Kyrylenko S., Henshel D., Yakymenko I. (2013). GSM 900 MHz cellular phone radiation can either stimulate or depress early embryogenesis in Japanese quails depending on the duration of exposure. Int. J. Radiat. Biol..

[24] Jing J., Zhang Y., Yang X., Jiang R., Guo D., Cui X. (2012). The influence of microwave radiation from cellular phone on fetal rat brain. Electromagn. Biol. Med..

[25] Yakymenko I., Sidorik E., Tsybulin O., Chekhun V. (2011). Potential risks of microwaves from mobile phones for youth health. Environ. Health.

[26] Bortkiewicz A. (2001). Research on the effectiveness of biological activities of EMF at frequencies emitted by mobile phones. Occup. Med..

[27] Gherardini L., Ciuti G., Tognarelli S., Cinti C. (2014). Searching for the perfect wave: The effect of radiofrequency electromagnetic fields on cells. Int. J. Mol. Sci..

[28] Adams J., Galloway T., Mondal D., Esteves S., Mathews F. (2014). Effect of mobile telephones on sperm quality: A systematic review and meta-analysis. Environ. Int..

[29] Varga K., Noisternig M.F., Griesser U.J., Alja L., Koch T. (2011). Thermal and Sorption Study of Flame Resistant Fibers. Lenzing. Berichte.

[30] Sowa P., Rutkowska-Talipska J., Sulkowska U., Rutkowski K., Rutkowski R. (2012). Electromagnetic radiation in modern medicine: Physical and biophysical properties. Pol. Ann. Med..

[31] Güler S., Yenikaya S., Yılmaz G. (2020). Shielding Effectiveness Analysis of Electronic Equipment Protection Box. Uludağ Univ. J. Fac. Eng..

[32] Gryz K., Karpowicz J., Kurczewska A., Stefko A., Smalcerz A. (2009). Reduction of occupational risk with electromagnetic sources—Review of selected barrier materials. Occup. Saf..

[33] Sengupta D.L., Liepa V.V. (2006). Applied Electromagnetics and Electromagnetic Compatibility.

[34] Kunkel G.M. Introduction to Shielding of Electromagnetic Fields and the Application to EMI/RFI Gaskets. Proceedings of the 1975 IEEE International Symposium on Electromagnetic Compatibility.

[35] Blachowicz T., Wojcik D., Surma M., Magnuski M., Ehrmann G., Ehrmann A. (2023). Textile Fabrics as Electromagnetic Shielding Materials-A Review of Preparation and Performance. Fibers.

[36] Pušić T., Saravanja B., Malarić K. (2021). Electromagnetic Shielding Properties of Knitted Fabric Made from Polyamide Threads Coated with Silver. Materials.

[37] Kaya D., Oglakcioglu N., Sarı B., Aktekeli Yılmaz H. (2023). Electromagnetic Shielding and Comfort Properties of Knitted Fabrics Produced by Electrically Conductive Fibers. Fibers Polym..

[38] Tunakova V., Tunák M., Bajzik V., Ocheretna L., Arabuli S., Kyzymchuk O., Vlasenko V. (2020). Hybrid knitted fabric for electromagnetic radiation shielding. J. Eng. Fibers Fabr..

[39] Yuzer A., Abdulla R., Erdem E., Abdulla F. (2017). Electromagnetic shielding characterization of conductive knitted fabrics. Prog. Electromagn. Res. M.

[40] Shieldex Site. https://www.shieldex.de/en/.

[41] Markek Site. https://marktek-inc.com/.

[42] Król I.A., Redlich G., Obersztyn E., Fortuniak K., Maklewska E., Olejnik M., Bartczak A. (2010). Raw Materials with Electrically Conductive Properties in Highly Specialized Products. http://archive.moratex.eu/pliki/tww/2010_34/TWW_2010_3-4.pdf.

[43] Tamburrano A., Desideri D., Maschio A., Sabrina Sarto M. (2014). Coaxial Waveguide Methods for Shielding Effectiveness Measurement of Planar Materials Up to 18 GHz. IEEE Trans. Electromagn. Compat..

[44] Wilson P.F. (1998). Techniques for measuring the electromagnetic shielding effectiveness of materials: Part I—Far-field source simulation. IEEE Trans. Electromagn. Compat..

[45] Wu Y., Zhous S., Xu Z., Yu W. Effect of Carbon Fiber Buckling Waved Arrangement on the Absorption of Electromagnetic Wave. Proceedings of the Asia Pacific Conference on Environmental Science and Technology Advances in Biomedical Engineering.

[46] Catrysse J.A., Delesie M., Steenbakkers W. (1992). The influence of the test fixture on shielding effectiveness measurements. IEEE Trans. Electromagn. Compat..

[47] Catrysse J.A. (1993). Correlation between shielding effectiveness measurements and alternative methods for the characterization of shielding materials. IEEE Trans. Electromagn. Compat..

[48] De Smedt R., Franchois A., Cumps M., Catrysse J.A. Circuit theory of the TEM-t with application to the measurement of the shielding effectiveness of thin, conductive materials. Proceedings of the International Symposium on Electromagnetic Compatibility.

[49] Bozzetti M., Pisu L., Sarto M., Greco S. Shielding performance of an expanded copper foil over a wide freqeuncy range. Proceedings of the 10th International Symposium on Electromagnetic Compatibility.

[50] (1983). Test Method for Electromagnetic Shielding Effectiveness of Planar Materials.

[51] (2010). Standard Test Method for Measuring the Electromagnetic Shielding Effectiveness of Planar Materials.

[52] Gooch J.W., Daher J.K. (2007). Fundamentals of Electromagnetic Shielding. Electromagnetic Shielding and Corrosion Protection for Aerospace Vehicles.

[53] Christopoulos C. (2022). Principles and Techniques of Electromagnetic Compatibility.

[54] Onuki H., Shinagawa M., Kajiya Y., Sato K. Using Electromagnetic Field Simulations to Evaluate the Impact of Complex Permeability on Shielding Effectiveness. Proceedings of the IEEE 12th Global Conference on Consumer Electronics (GCCE).

